# Estimation of Degradation Degree in Road Infrastructure Based on Multi-Modal ABN Using Contrastive Learning

**DOI:** 10.3390/s23031657

**Published:** 2023-02-02

**Authors:** Takaaki Higashi, Naoki Ogawa, Keisuke Maeda, Takahiro Ogawa, Miki Haseyama

**Affiliations:** 1 Graduate School of Information Science and Technology, Hokkaido University, N-14, W-9, Kita-ku, Sapporo 060-0814, Hokkaido, Japan; 2 Faculty of Information Science and Technology, Hokkaido University, N-14, W-9, Kita-ku, Sapporo 060-0814, Hokkaido, Japan

**Keywords:** self-supervised learning, contrastive learning, distress image classification, convolutional neural network, multi-modal learning

## Abstract

This study presents a method for distress image classification in road infrastructures introducing self-supervised learning. Self-supervised learning is an unsupervised learning method that does not require class labels. This learning method can reduce annotation efforts and allow the application of machine learning to a large number of unlabeled images. We propose a novel distress image classification method using contrastive learning, which is a type of self-supervised learning. Contrastive learning provides image domain-specific representation, constraining such that similar images are embedded nearby in the latent space. We augment the single input distress image into multiple images by image transformations and construct the latent space, in which the augmented images are embedded close to each other. This provides a domain-specific representation of the damage in road infrastructure using a large number of unlabeled distress images. Finally, the representation obtained by contrastive learning is used to improve the distress image classification performance. The obtained contrastive learning model parameters are used for the distress image classification model. We realize the successful distress image representation by utilizing unlabeled distress images, which have been difficult to use in the past. In the experiments, we use the distress images obtained from the real world to verify the effectiveness of the proposed method for various distress types and confirm the performance improvement.

## 1. Introduction

The average length of public roads is more than 500,000 km in OECD countries [1]. These road infrastructures are reported to be aging [2,3]. The maintenance management for aging road infrastructures is increasingly becoming essential in preventing fatal incidents. After acquiring distress images and other information obtained from close-up visual inspection, palpation, and hammering, the degree of degradation levels is determined by skilled engineers during road infrastructure inspections [4]. Meetings are specifically held with several engineers, and the degradation degree is determined using on-site inspection results, including taken distress images. However, an enormous amount of labor is required considering the large number of distress images to be checked. This requires techniques that can reduce the burden on engineers.

Various techniques have been proposed to support engineers using supervised deep learning [5,6,7,8,9,10]. Studies in [5,6,7] have constructed methods based on the convolutional neural network (CNN) [11] that achieves high performance in generic object recognition. Researchers have actively attempted to construct CNN-based supervised models that can automatically inspect distresses in images [5,6,7]. Other studies [8,9,10] have constructed methods based on the attention branch network (ABN) [12] to solve the problem encountered by many supervised learning models, including CNN, that is, the failure to determine the reason for the classification results. ABN has a CNN-based backbone and generates an attention map that indicates the regions of interest in distress images. For road infrastructure distresses, ABN-based methods improve the estimation performance of the degradation degree considering the highlighted regions [8,9,10]. Supervised classification methods, such as ABN, were originally proposed for classification in the field of general object recognition, in which a large-scale training dataset, such as ImageNet [13], is available. Classification methods require a large number of images and class label pairs for stable training. However, due to the burden of skilled engineers with respect to annotating degradation degrees, preparing many distress images with class labels is more difficult than preparing images used in general object recognition. Achieving a high performance using supervised learning models is not realistic in these settings.

Self-supervised learning, which does not require class labels, attracted much attention in the field of deep learning [14,15,16,17]. Contrastive learning, a type of self-supervised learning, particularly enables the acquisition of domain-specific representation from many unlabeled images [15,16,17]. Contrastive learning is performed in advance, and the obtained domain-specific representation is used to improve the classification performance based on supervised learning. Specifically, in contrastive learning, each image in the dataset is first augmented using several transformations, such as cropping and changing colors. To capture the image dataset features, a latent space is then constructed in the deep learning model based on the similarity using the augmented images. The obtained model parameters can be used as the initial parameters for training various supervised learning models. A high classification performance can be achieved by using prior representation from unlabeled images, even when the number of training images is limited for supervised learning [15,16,17]. Contrastive learning has been introduced in various fields, such as remote sensing [18,19], point cloud analysis [20,21], and medical science [22,23], with a high burden for annotation. In particular, the final classification performance can be improved by using the model parameters obtained through contrastive learning for a small sample dataset of medical images with unique characteristics compared to general images [22,23]. In road infrastructures, distress images mainly capture construction materials, such as concrete. Similar to medical images, distress images have a more unique domain than general images. Therefore, contrastive learning is expected to be used in comprehending distresses using many images without labels. Contrastive learning can improve the distress image classification performance via the ABN-based method using the prior representation.

In this paper, we propose a novel distress image classification method for road infrastructures using contrastive learning. First, a CNN-based model is trained by contrastive learning from unlabeled distress images. Next, the image classification model is trained for each distress, such as crack and efflorescence. Contrastive learning is performed by using multiple distress types. The general representation is obtained for road infrastructure distresses. As the representation obtained by contrastive learning, the tuned CNN parameters can be introduced into the classification model based on ABN. In our method, the ABN-based model uses not only distress images but also corresponding text for correcting attention maps and estimating the degradation degrees. However, the use of text data is not sufficient in reinforcing the ability to extract distress image representations. Thus, the CNN-based parameters obtained through contrastive learning are utilized. Then, we achieve the effective extraction of image representations by CNN layers, such as feature maps. This is expected to improve the performance. In summary, the contributions of our method are shown below.

- By introducing contrastive learning, we realize the estimation of the degradation degree of distress images using not only labeled but also unlabeled training data to reduce the annotation burden of engineers.
- We realize the acquisition of general representation specific to damaged road infrastructures and the improvement of the estimation performance specializing in each distress.

The remainder of this paper is organized as follows. Section 2 presents the related works on distress image classification based on supervised and contrastive learning. Section 3 introduces the proposed estimation method of degradation degrees based on contrastive learning to utilize unlabeled distress images, which have been difficult to use in the past. Section 4 presents the experiments, and Section 5 provides the conclusion.

## 2. Related Works

To clarify the contribution and novelty of our method for supporting engineers in real-world applications, this section presents distress image classification based on supervised and contrastive learning.

### 2.1. Distress Image Classification Based on Supervised Learning

Aiming at real-world applications, various deep learning methods have been introduced for tasks in the civil engineering field. The main purpose of those methods is the streamlining inspection operations to detect damages in infrastructures for preventing serious accidents. In previous studies [5,6,7,24,25,26,27,28,29], models were built to detect distresses in the taken image. In particular, the studies [7,25,28,29] attempted to visualize the corresponding regions of cracks in distress images as the segmentation task. The studies [5,6,7,24,25,26,27,28,29] achieved their tasks by extracting the features of distress images by introducing CNNs. Other previous studies [5,8,9,10,30] target the classification of distresses (e.g., crack, efflorescence or corrosion) that have occurred in infrastructures. In particular, the studies [8,9,10] attempted to estimate the degree of degradation in road infrastructures. The estimation of the degradation degrees leads to a judgment of the distress which should be repaired as a high priority and the realization of efficient field operations. In addition, refs. [8,9,10] introduced an attention mechanism that enables the classification model to emphasize important regions in the distress image. The model based on the mechanism makes it possible to provide engineers with a basis for judging the degree. The technique is not expected to fully automate the judgment of degrees but can be applied as a supplemental tool for engineers to judge.

The above studies [5,6,7,8,9,10,24,25,26,27,28,29,30] constructed supervised learning models by using the label information of images as ground truth. For general object recognition in the field of computer vision, the performance improvement is rapid, and the contribution of large image datasets, such as ImageNet [13] and Microsoft common object in context [31], has been significant. Unlike general images, the specialized skill of engineers is essential for the annotation process of distress images. The training is conducted with a small number of pairs in civil engineering. Thus, as one of the training measures, the initial parameters of the models in the studies [5,8,9,10] are set to those obtained from a general image dataset. However, since the characteristics of general and distress images are quite different, using parameters based on a general image dataset is not optimal. If we can use parameters obtained from many unlabeled distress images as the initial parameters of supervised learning, we expect to improve the performance of the target task even with supervised learning by using a small number of samples. Thus, the introduction of unsupervised learning approaches is an effective solution to this problem.

### 2.2. Contrastive Learning for Distress Image Classification

Contrastive learning is a self-supervised learning technique of acquiring informative representations from the data themselves by focusing on their similarity. Unlike supervised learning, self-supervised learning can use unlabeled training data and does not necessarily require a great deal of human effort for annotation. Thus, self-supervised learning has been actively introduced into various fields, such as computer vision [15,16,17,32], natural language processing [33,34], speech recognition [35,36], remote sensing [18,19], point cloud analysis [20,21], and medical science [22,23]. In the field of computer vision [15,16,17], contrastive learning can construct a latent space in which similar images are embedded close to each other. When each sample is taken from a mini-batch (hereafter denoted as an anchor), the image transformations are applied to generate an augmented version of each sample (hereafter denoted as a view). From anchors and generated views, the objective function of contrastive learning is defined to obtain the same representations between similar data. In many methods, contrastive learning is performed such that the anchor and the view generated from the same anchor (hereafter denoted as a positive sample) are embedded close together in the latent space. Depending on the type of contrastive learning, views with different characteristics from the anchor (hereafter denoted as negative samples) are embedded farther apart in the latent space.

Contrastive learning can construct CNN-based models and is highly compatible with the previous methods [5,6,7,8,9,10,24,25,26,27,28,29,30] to utilize the convolution of images. The introduction of contrastive learning enables the use of the knowledge obtained from previous studies in the civil engineering field. Thus, contrastive learning is one of the solutions to the problem of labeling difficulty, which is a fundamental difference between general and distress images. If the labeling process is omitted, the burden on engineers can be reduced, and inspection work becomes more efficient. Furthermore, many conservation organizations have taken distress images in their inspection work. Since contrastive learning without labels makes it feasible to utilize many distress images taken by different organizations, it facilitates the collection of the images needed for training.

Contrastive learning has been implemented for railway maintenance, as an application similar to our target field [37]. The previous study attempted to classify distress types, such as crack and spalling. In addition, this study targeted the metal and wood of the rails, which are very different from road infrastructures whose main material is concrete. Efflorescence and rebar corrosion within concrete are also detected in the distresses that occurred on road infrastructures, and these types of distress can lead to serious accidents. It is necessary to sufficiently validate the comprehensive effectiveness of introducing contrastive learning for various distress types in the real world.

## 3. Estimation of Degradation Degree in Road Infrastructure Using Contrastive Learning

**Figure 1:**
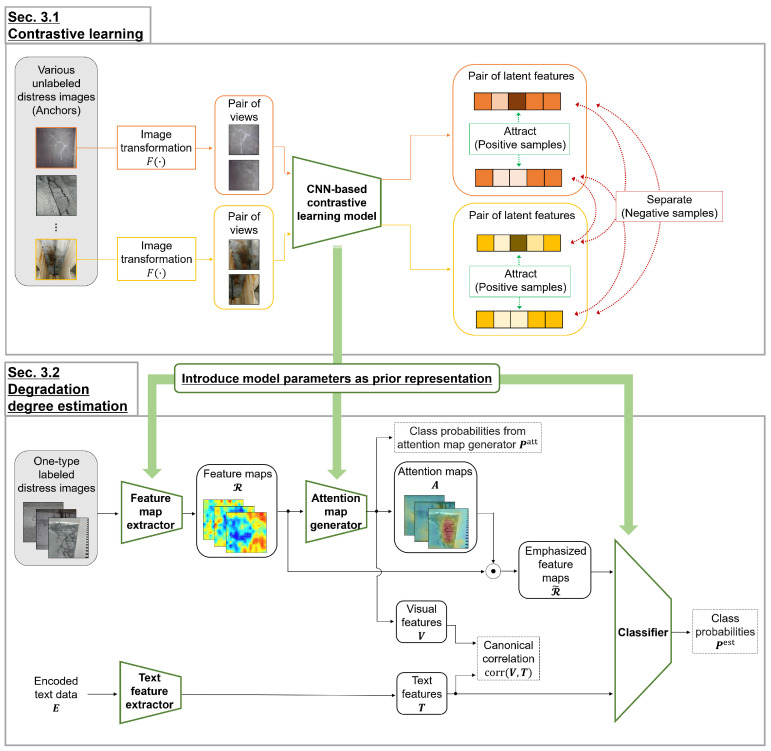
Overview of the proposed method in the training phase. Contrastive learning corresponds to Section 3.1 and is performed using CNN as the backbone. Augmented images, called views, are generated via image transformation F(·) from original images, called anchors. The latent space is constructed such that views from the same anchor are close to each other, and the others are separated from each other. Estimating degradation degrees of distress images corresponds to Section 3.2. The classification model is constructed based on ABN, which has the same CNN backbone of contrastive learning. As the initial parameters of the classification model, those obtained by contrastive learning are used. The parameters of contrastive learning are specifically set to the feature map extractor, attention map generator, and classifier in the model. In supervised learning for estimating the degradation degree, the classification model is trained using both images and their corresponding encoded text data E. The correct regions are emphasized in the attention map by using the canonical correlation calculated between the visual features V and text features T.

This section explains the proposed method. Figure 1 illustrates an overview of the training phase. First, contrastive learning is performed on the CNN-based model by using unlabeled distress images. Next, the ABN-based model is trained to estimate the degradation degree of each distress type through supervised learning. The ABN-based model has the same CNN backbone in our contrastive learning. Section 3.1 and Section 3.2 present the representation acquisition through contrastive learning and the estimation of degradation degrees using the prior representation, respectively.

### 3.1. Representation Acquisition through Contrastive Learning

Contrastive learning is performed by using multiple type distress images to construct the latent space that captures the distress properties of road infrastructures. Then, the parameters obtained through contrastive learning are used for the following supervised learning to estimate the degradation degree.

We use SimCLR [17] whose backbone is CNN, ResNet50 [38], as the contrastive learning method. SimCLR augments images, called views, by applying the image transformation F(·) that randomly combines cropping, color change, and Gaussian noise addition to the training image as follows:

(1) $$\begin{array}{cc} x_{i},x_{j} & =F(x), \end{array}$$

where *x* is an input image, called anchor, and $x_{i}$ and $x_{j}$ are the generated views. The image transformation F(x) generates two views for each anchor. A total of 2N views are generated for *N* samples. SimCLR calculates the similarity between the generated views. In the two views generated from the same anchor, one view is defined as the target image, and the other is defined as the positive sample. The remaining 2(N−1) views are used as negative samples. With view *i* as the target and the other *j* as the positive sample, the model in SimCLR is trained based on the loss function $L_{i,j}$ in the following equation:

(2) $$\begin{array}{cc} L_{i,j} & =-\log\frac{\exp\left(\mathrm{sim}\left(h_{i},h_{j}\right)/\tau \right)}{\sum_{n=1}^{2N}O_{\left[n\neq i\right]}\exp\left(\mathrm{sim}\left(h_{i},h_{n}\right)/\tau \right)}, \end{array}$$

where $O_{\left[n\neq i\right]}\in \left\{0,1\right\}$ is 1 for n≠i and 0 for n=i, $h_{i}\in R^{d_{r}}$ is the feature representation of image *i* in the latent space (n=1,⋯,2N, and $d_{r}$ being the dimension of the feature representation), and τ is a temperature parameter. $\mathrm{sim}\left(a,b\right)=a^{⊤}b/∥a∥\;∥b∥$. According to Equation (2), the latent space is constructed based on the calculated similarity, such that the loss of a positive sample pair is large, and the losses of the negative sample pairs are small. Each generated view is used as a pseudo-supervisory label, and we can construct the latent space from an unlabeled distress image dataset. Finally, the proposed method can obtain the model parameters as the prior properties of the distress images in road infrastructures.

### 3.2. Degradation Degree Estimation Using Prior Representation

**Figure 2:**
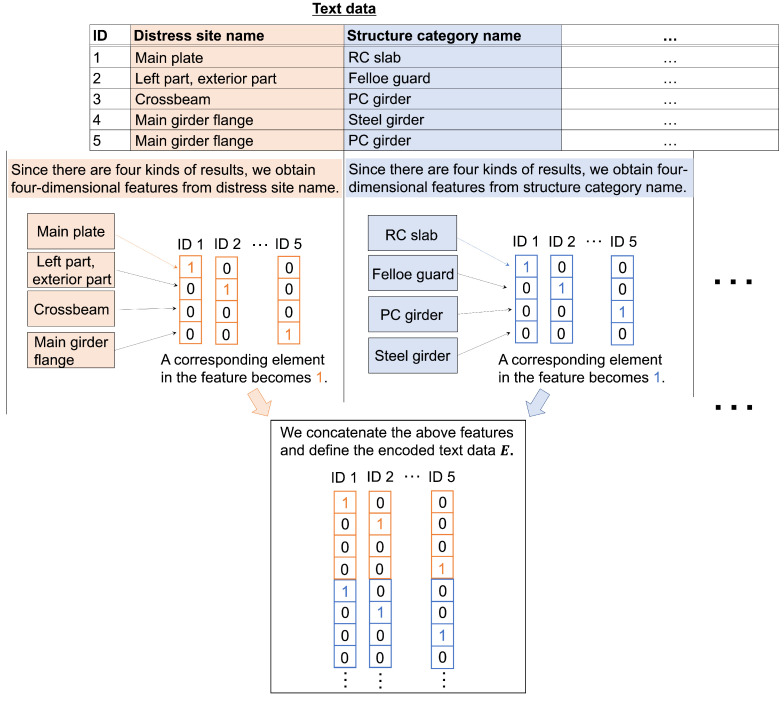
Details of the encoding approach for the text data. In the case of M=5, each sample is assigned to IDs 1 to 5. As shown in “Distress site name,” there are four kinds of inspection results; hence, we obtain four-dimensional features for this item. ID1 has a main plate as “Distress site name”, and the corresponding element in the feature becomes 1, and the other elements become 0. Finally, the obtained features are concatenated for each ID, and the encoded text data E are defined. This encoding approach is based on the studies [30].

We describe estimating degradation degrees of distress images in road infrastructures using the prior representation obtained through contrastive learning. We use the correlation-aware attention branch network (CorABN) proposed in the study [8]. This model has the attention mechanism, which emphasizes the important regions of the feature maps. CorABN also extracts text features from text data including location and structure name corresponding to the distress images. The text data are encoded as vectors with multiple one-hot vectors concatenated as $E=\left[e_{1},\cdots ,e_{M}\right]\in R^{d_{e}\times M}$ ($d_{e}$ being the dimension of the encoded text data and *M* being the number of samples). Figure 2 presents the encoding details. Unlike the simple ABN, the model improves the estimation performance by introducing the attention mechanism that uses text data to generate attention maps.

CorABN has a backbone based on ResNet50 consisting of a simple CNN layer and four residual blocks in order from the input. Thus, it can share the model parameters obtained from SimCLR of the same backbone. In Figure 1, the feature map extractor sets the parameters of the CNN layer and the following first to third residual blocks, while the attention map generator and the classifier set the parameters of the fourth residual block. Then, we realize the effective use of the prior representations obtained by contrastive learning in CorABN. CorABN estimates the degradation degree considering the important regions in the attention map. The generated attention map emphasizes the feature maps as follows:

(3) $$\begin{array}{c} {\tilde{R}}_{m,l}=\left(1+A_{m}\right)⊙R_{m,l}, \end{array}$$

where $A_{m}\in R^{d_{H}\times d_{W}}$ is an attention map (m=1,…,M, and $d_{H}$ and $d_{W}$ being the dimensions in the attention map corresponding to the height and width), ⊙ is the Hadamard product, and $R_{m}=\left[R_{m,1},\cdots ,R_{m,d_{L}}\right]\in R^{d_{L}\times d_{H}\times d_{W}}$ is a tensor composed of feature maps ($l=1,\ldots ,d_{L};$$d_{L}$ being the number of channels in the feature maps). ${\tilde{R}}_{m}=\left[{\tilde{R}}_{m,1},\cdots ,{\tilde{R}}_{m,d_{L}}\right]\in R^{d_{L}\times d_{H}\times d_{W}}$ is a tensor composed of the emphasized feature maps calculated using the attention map $A_{m}$ and feature maps $R_{m}$. Note that $\tilde{R}\in \left\{{\tilde{R}}_{1},{\tilde{R}}_{2},\cdots ,{\tilde{R}}_{M}\right\}$, $A\in \{A_{1},A_{2},\cdots ,A_{M}\}$, and $R\in \{R_{1},R_{2},\cdots ,R_{M}\}$ are represented as a group of *M* samples in Figure 1.

The loss function $L_{\mathrm{CorABN}}$ is defined based on the class probabilities $P^{\mathrm{est}}=\left[p_{1}^{\mathrm{est}},\cdots ,p_{M}^{\mathrm{est}}\right]\in R^{d_{C}\times M}$ ($d_{C}$ being the number of classes). The canonical correlation $L_{\mathrm{cor}}\left(V,T\right)=-\mathrm{corr}\left(V,T\right)$ is calculated between the visual features $V=\left[v_{1},\cdots ,v_{M}\right]\in R^{d_{V}\times M}$ ($d_{V}$ being the dimension of the visual features) and text features $T=\left[t_{1},\cdots ,t_{M}\right]\in R^{d_{T}\times M}$ from the encoded text data E ($d_{T}$ being the dimension of the text features). The text features T are extracted by the text feature extractor in Figure 1 based on a multilayer perceptron. From the above, the loss function $L_{\mathrm{CorABN}}$ is defined as follows:

(4) $$\begin{array}{c} L_{\mathrm{CorABN}}=L_{\mathrm{CE}}\left(\mathrm{GT},P^{\mathrm{att}}\right)+\beta \cdot L_{\mathrm{CE}}\left(\mathrm{GT},P^{\mathrm{est}}\right)+\gamma \cdot L_{\mathrm{cor}}\left(V,T\right), \end{array}$$

where $\mathrm{GT}=\left[{\mathrm{gt}}_{1},\cdots ,{\mathrm{gt}}_{M}\right]\in R^{d_{C}\times M}$ is the one-hot vector, in which only one element corresponding to the correct degradation degree is set to 1, and the others are set to 0. $P^{\mathrm{att}}=\left[p_{1}^{\mathrm{att}},\cdots ,p_{M}^{\mathrm{att}}\right]\in R^{d_{C}\times M}$ is the class probabilities from the attention map generator, β and γ are the hyperparameters, and $L_{\mathrm{CE}}$ is the cross-entropy loss. For example, if the true class probability distribution is $P^{\prime }=\left[{p^{\prime }}_{1},\cdots ,{p^{\prime }}_{M^{\prime }}\right]\in R^{d_{C^{\prime }}\times M^{\prime }}$, and the estimated class probability distribution is $Q^{\prime }=\left[{q^{\prime }}_{1},\cdots ,{q^{\prime }}_{M^{\prime }}\right]\in R^{d_{C^{\prime }}\times M^{\prime }}$ ($d_{C^{\prime }}$ and $M^{\prime }$ being the numbers of classes and samples), $L_{\mathrm{CE}}\left(P^{\prime },Q^{\prime }\right)$ is defined as follows:

(5) $$\begin{array}{c} L_{\mathrm{CE}}\left(P^{\prime },Q^{\prime }\right)=-\sum_{m^{\prime }=1}^{M^{\prime }}\sum_{c^{\prime }=1}^{C^{\prime }}{p^{\prime }}_{m^{\prime },c^{\prime }}\log{q^{\prime }}_{m^{\prime },c^{\prime }}. \end{array}$$

$L_{\mathrm{cor}}$ in Equation (4) is inspired by the study [39]. For example, if ${Z^{\prime }}_{1}\in R^{d_{Z^{\prime }}\times M^{\prime }}$ and ${Z^{\prime }}_{2}\in R^{d_{Z^{\prime }}\times M^{\prime }}$ ($d_{Z^{\prime }}$ being the dimension of $Z^{\prime }$) are defined as the matrices encoded by the deep neural network models, $L_{\mathrm{cor}}\left({Z^{\prime }}_{1},{Z^{\prime }}_{2}\right)$ is calculated as follows:

(6) $$\begin{array}{cc} L_{\mathrm{cor}}\left({Z^{\prime }}_{1},{Z^{\prime }}_{2}\right) & =-\mathrm{corr}({Z^{\prime }}_{1},{Z^{\prime }}_{2})\\ \; & =-\left|\right|\Sigma_{11}^{-1/2}\Sigma_{12}\Sigma_{22}^{-1/2}{\left|\right|}_{\mathrm{tr}}, \end{array}$$

where $\Sigma_{11}=\frac{1}{M^{\prime }-1}\tilde{{Z^{\prime }}_{1}}{\tilde{{Z^{\prime }}_{1}}}^{⊤}+r_{1}I$ ($\tilde{{Z^{\prime }}_{1}}$ being the centered data matrix from ${Z^{\prime }}_{1}$, $r_{1}$ being a parameter for the regularization constraint, and I being an identity matrix), $\Sigma_{12}=\frac{1}{M^{\prime }-1}\tilde{{Z^{\prime }}_{1}}{\tilde{{Z^{\prime }}_{2}}}^{⊤}$ ($\tilde{{Z^{\prime }}_{2}}$ being the centered data matrix from ${Z^{\prime }}_{2}$), and $\Sigma_{22}=\frac{1}{M^{\prime }-1}\tilde{{Z^{\prime }}_{2}}{\tilde{{Z^{\prime }}_{2}}}^{⊤}+r_{2}I$ ($r_{2}$ being a parameter for the regularization constraint). Assume that $r_{1}>0$, such that $\Sigma_{11}$ is positive definite (resp. $r_{2}$).

Using the loss function $L_{\mathrm{CorABN}}$ in Equation (4), CorABN can be trained to correct the attention map in the region of interest using the text data. CorABN extracts feature and attention maps from distress images and estimates the degradation degree to use the emphasized feature maps. Thus, the calculations for these maps are important through the CNN layers in CorABN. In Figure 1, the feature map extractor, attention map generator, and classifier can share with the parameters of the CNN-based contrastive learning model from many unlabeled images, regardless of the distress types. In the classification for each distress type, the degradation degree is estimated by the proposed method, which effectively uses not only the text data but also the parameters of contrastive learning as the prior general representation.

## 4. Experimental Results and Discussion

This section presents the experimental results of estimating degradation degrees of distress images. Section 4.1–Section 4.4 provide the experimental settings, results, discussions, and limitations and future work, respectively.

### 4.1. Experimental Settings

We used two datasets consisting of the distress images in the road infrastructures provided by East Nippon Expressway Company Limited to train the proposed method. The first dataset, called the unlabeled dataset, includes various images not limiting the distress types. The second dataset, called the labeled dataset, includes the distress images annotated with the degradation degree and their corresponding text data. The images in both datasets were resized to 224 × 224 px.

Contrastive learning was performed for the unlabeled dataset containing 168,315 distress images of road infrastructures for acquiring the prior representation. The dimension $d_{r}$ of the feature representations was set to 64 in the loss function *L* of Equation (2). The learning rate was set to 0.0003. The batch size was set to 128. The temperature parameter τ was set to 0.5. The number of epochs was grid-searched every 10 epochs up to 200 epochs. The model parameters were selected based on the highest estimation performance. The computations were performed on a computer with an Intel(R) Core(TM) i9-10980XE CPU @ 3.00 GHz, 128.0 GB of RAM, and a single TITAN RTX GPU.

**Figure 3:**
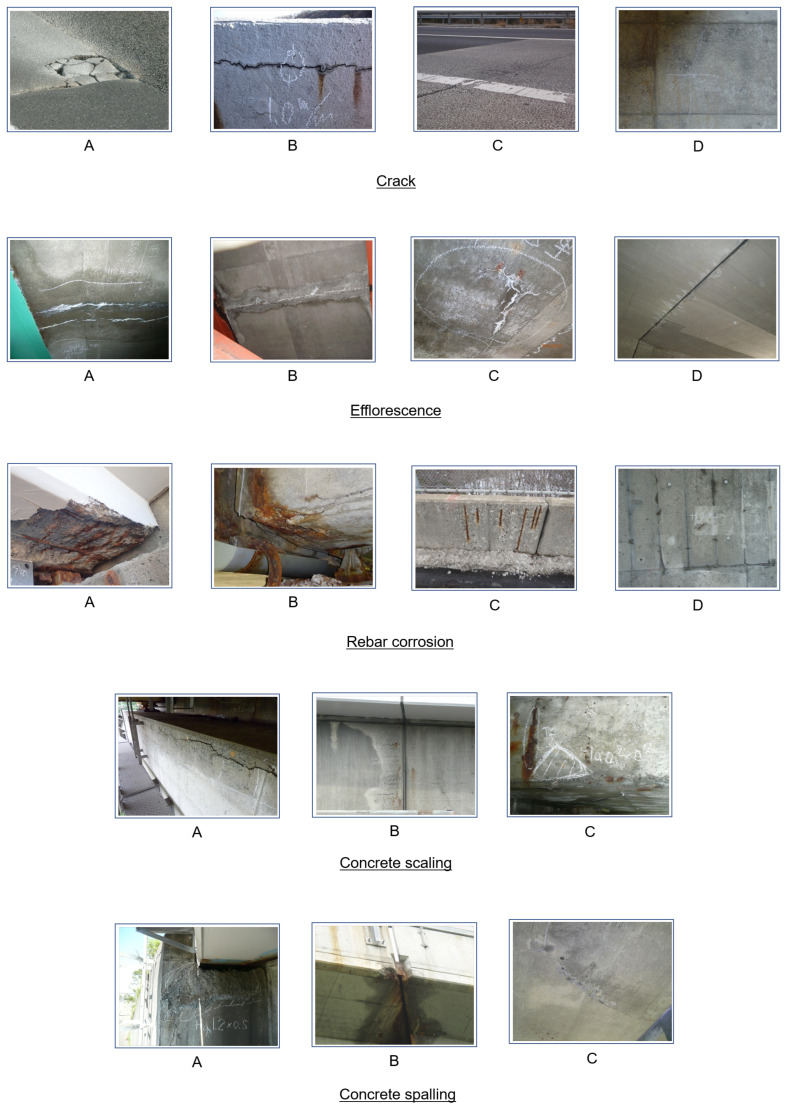
Examples of each degradation degree in the types of crack, efflorescence, rebar corrosion, concrete scaling, and concrete spalling. Crack, efflorescence, and rebar corrosion were evaluated at four degrees, and concrete scaling and spalling were evaluated at three degrees by skilled engineers.

Supervised learning in CorABN was performed from the labeled dataset. Table 1, Table 2, Table 3, Table 4 and Table 5 show the degradation degrees and the numbers of images for the training, validation, and test. The dataset consisted of the distress images of crack, efflorescence, rebar corrosion, concrete scaling, and concrete spalling with the annotated degradation degrees. The types of crack, efflorescence, and rebar corrosion were classified by four-stage degradation degrees from A to D in order of increasing danger. The rest of the types, concrete scaling and spalling, were classified by three-stage degradation degrees from A to C. These degradation degrees were used as the ground truth in the supervised learning and explained in detail in [40]. Figure 3 shows examples of each distress image. The text data included 10 types of items: “Distress item name”, “Distress category name”, “Distress type name”, “Distress site category name”, “Distress site name”, “Branch code”, “Office code”, “Structure type name”, “Structure category name”, and “Inspection method name”. The dimension of the encoded text data $d_{e}$ is different for each distress type. For crack, efflorescence, rebar corrosion, concrete scaling, and spalling, $d_{e}$ was 223, 160, 187, 186, and 179, respectively. CorABN models were separately trained to estimate the degradation degree for each distress type. The text feature extractor was a module based on a 5-layer multi-layer perceptron (MLP). The dimensions of the 3-intermediate and 1-output layers were set to 64, 32, 16, and 8. The dimensions of the input layers were set to $d_{e}$ for each distress type. The texts assigned to each distress type were different and the dimensions of the input layer were also different. In addition, we used the sigmoid function as the activation of MLP. Following the study [8], the hyperparameters β and γ were set to 1.0 and 0.2, respectively. The batch size was set to 16. The learning rate was set to 0.01 at the beginning and decreased by 1/10 for every 10 epochs. The CorABN model parameters were grid-searched from 1 to 30 epochs. The epoch was selected based on the highest estimation performance from the validation data. The computational environment was the same as that used in our contrastive learning.

F1-score was used as the evaluation metric of the estimation performance. F1-score is the harmonic mean of Precision and Recall presented as follows:

(7) $$\begin{array}{c} F1-\mathrm{score}=\frac{2\times \mathrm{Precision}\times \mathrm{Recall}}{\mathrm{Precision}+\mathrm{Recall}}, \end{array}$$

where

(8) $$\begin{array}{c} \mathrm{Precision}=\frac{\mathrm{Number}\;\mathrm{of}\;\mathrm{images}\;\mathrm{whose}\;\mathrm{degree}\;\mathrm{was}\;\mathrm{correctly}\;\mathrm{estimated}\;\mathrm{as}\;\mathrm{each}\;\mathrm{degree}}{\mathrm{Number}\;\mathrm{of}\;\mathrm{images}\;\mathrm{estimated}\;\mathrm{as}\;\mathrm{each}\;\mathrm{degree}}, \end{array}$$

(9) $$\begin{array}{c} \mathrm{Recall}=\frac{\mathrm{Number}\;\mathrm{of}\;\mathrm{images}\;\mathrm{whose}\;\mathrm{degree}\;\mathrm{was}\;\mathrm{correctly}\;\mathrm{estimated}\;\mathrm{as}\;\mathrm{each}\;\mathrm{degree}}{\mathrm{Number}\;\mathrm{of}\;\mathrm{all}\;\mathrm{images}\;\mathrm{annotated}\;\mathrm{with}\;\mathrm{each}\;\mathrm{degree}}. \end{array}$$

F1-score takes values from 0 to 1. A higher value means higher performance.

We evaluated the effectiveness of the proposed method (PM) using several comparison methods (CMs). We performed the degradation degree estimation using only CorABN proposed in the study [8]. A comparison of CorABN with PM verified the effectiveness of introducing the prior representation obtained by contrastive learning. ABN [12] based on ResNet50 [38] was used in the experiment because it is a baseline method in image classification. The only difference between CorABN and ABN is the use of text data. We also compared PM with multiple CNNs, ResNet50 [38], SeNet154 [41], InceptionV4 [42], DenseNet121 [43], and EfficientNetB5 [44], to confirm the effectiveness of PM. In this experiment, we conducted 10 runs for each distress and evaluated the estimation performances of PM and CMs, calculating the F1-score averages.

### 4.2. Experimental Results

#### 4.2.1. Quantitative Evaluation

Table 6, Table 7, Table 8, Table 9 and Table 10 show F1-scores for the estimation of degradation degrees corresponding to crack, efflorescence, rebar corrosion, concrete scaling, and spalling, respectively. Table 6 and Table 8, Table 9 and Table 10 of crack, rebar corrosion, concrete scaling, and spalling illustrate that the PM’s performance is better than that of CMs for all degradation degrees. Table 7 of efflorescence illustrates that the PM scores exceed not only those of CMs in half of the degrees but also the average. These results confirm that PM can achieve an improved estimation performance for many degradation degrees. PM contributes to improving overall performance.

We verified statistical superiority by Welch’s *t*-test. The t-test was performed on the average F1 scores for all degradation degrees between PM and CorABN [8], which does not use contrastive learning. For the four distress types of crack, rebar corrosion, concrete scaling, and spalling, the *p*-values were 0.02033, 0.00022, 0.00039, and 0.00391, respectively. The results confirmed the superiority of PM with *p*-values less than 0.05 at the significance level. In efflorescence, the result confirmed the superiority of PM with the *p*-value 0.29919 less than 0.3. The above t-test results indicate the statical effectiveness of the introduction of contrastive learning, which is the novelty of PM.

Among the five distress types, the most unbalanced dataset is rebar corrosion in Table 3. In this case, the number of samples for each degree significantly influences the classification. Specifically, in Table 8, degree B has the highest number of samples among the four-stage degrees and achieves the highest estimation performance. Degree A has the lowest number of samples and showed the lowest estimation performance. On the other hand, degree C, which has the second largest number of data, has lower performance than degree D, which has a smaller number of samples than degree C. This result was likely caused due to the order of degradation degrees that makes it difficult to estimate intermediate degrees such as B and C. Focusing on the remaining four distress types, Table 1, Table 2, Table 4 and Table 5 show that the dataset for the four types is not relatively unbalanced compared to rebar corrosion. In these cases, we confirmed the tendency that the higher estimation performances are achieved in the degrees of the greatest risk and the least risk as shown in Table 6, Table 7, Table 9 and Table 10. In conclusion, if the unbalanced dataset is used, the experimental result suggests that both the number of samples in each degree and the ordinality of the degrees affect the estimation performance. In the relatively balanced case, the experiment suggests that the degree of ordinality becomes a large effect.

#### 4.2.2. Contribution of Each Module in CorABN including Contrastive Learning

We verify the compatibility of the parameters of contrastive learning with each module in CorABN. To confirm which module benefits from contrastive learning, we evaluated the estimation performance by selecting modules using the parameters of contrastive learning as initial values. CorABN has three modules, the feature map extractor (FME), attention map generator (AMG), and classifier (CF). In the experiment, the parameters of contrastive learning were set only for one of them or any two of them, and we used ImageNet-based parameters for the remaining modules following the study [8]. As the distress type, we used rebar corrosion whose performance improvement is significant.

Table 11 shows the results of eight methods, including the cases where the parameters of contrastive learning were introduced into all modules and the case where the ImageNet parameters were introduced [8]. We confirm that FME most contributes to performance improvement given the parameters of contrastive learning introduced into one or two of all modules. Compared to the case of all modules based on the ImageNet parameters, the above cases including FME achieve higher estimation performances. FME is a module for extracting feature maps, which are then used as the input of AMG and CF. Furthermore, contrastive learning aims to capture the features of distress images and is highly compatible with the feature map extraction. Thus, it is reasonable to infer that introducing the parameters of contrastive learning is the most effective for FME to improve the performance. Compared to the above six ablation experiments by using the parameters in one or two of all modules, the case of introducing parameters into all modules shows the best performance for most degradation degrees and their averages. These ablation experiments indicate that PM introducing the parameters of contrastive learning into all modules is effective.

#### 4.2.3. Effectiveness of Contrastive Learning Approach Using Another Model

This section verifies the further effectiveness of the contrastive learning by using another model. In this experiment, we used bootstrap your own latent (BYOL) [45]. Unlike SimCLR, BYOL is a contrastive learning model that does not use negative samples. SimCLR consists of a single CNN-based encoder using negative samples for the training, while BYOL has two asymmetric CNN-based encoders named an online network and a target network instead of using negative samples. Specifically, the prediction head is added to only the online network, and the target network provides the regression targets. The target network is trained based on exponential moving averages of the online network parameters, and the online network is trained to predict the output of the target network. It has been reported that BYOL achieves highly accurate classification in settings with relatively small batch sizes.

Table 12 shows the results of the degradation degree estimation of CorABN using the parameters obtained by SimCLR and BYOL (two types of PM), and ImageNet parameters [8], respectively. First, Table 12 shows that PM using the SimCLR parameters achieves high estimation performance. The obvious difference between BYOL and SimCLR is the use of negative samples, and BYOL has a more complex model than SimCLR. The experimental results indicate that a simple contrastive learning model with negative samples may be effective for using distress images that have unique characteristics compared to general images. On the other hand, for efflorescence, PM using the BYOL parameters achieves better performance than PM using SimCLR parameters. This result indicates that PM is likely to achieve further performance improvement by refining the contrastive learning approach in the future. Next, compared with PM using the BYOL parameters and CorABN using the ImageNet parameters, the result shows that PM using BYOL improves the performance in most degradation degrees and their averages. These results confirm the effectiveness of PM based on contrastive learning models and indicate the usefulness of the contrastive learning approach for estimating the degradation degree of distress images in road infrastructures.

#### 4.2.4. Robustness Corresponding to Varying Parameter of Contrastive Learning Model

This section verifies the robustness of PM by changing the parameter of the contrastive learning model. We have confirmed the effect of varying the dimension of the feature representation $d_{r}$ in SimCLR. We adopted dimensions 64, 128, and 256. Table 13 shows the results of the degradation degree estimation with the three kinds of dimensions. Comparing three PMs with varying dimensions, we cannot confirm a clear decrease in performance with increasing dimensions $d_{r}$. The results show the robustness of PM. In addition, the appropriate dimensions with the highest estimation performance differ for each distress type. Therefore, it is expected to achieve further performance improvements by searching for the appropriate dimensions. This may be an issue for future work.

#### 4.2.5. Qualitative Evaluation

**Figure 4:**
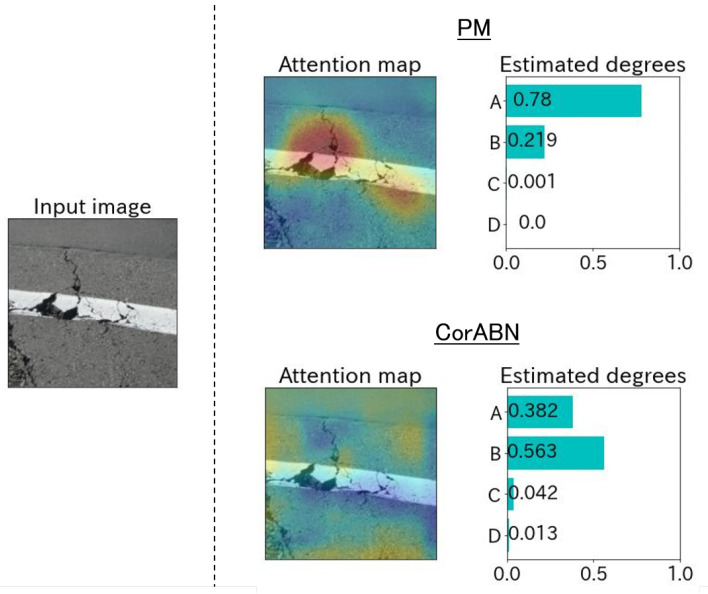
Estimated example at the degradation degree A for the distress type of crack.

**Figure 5:**
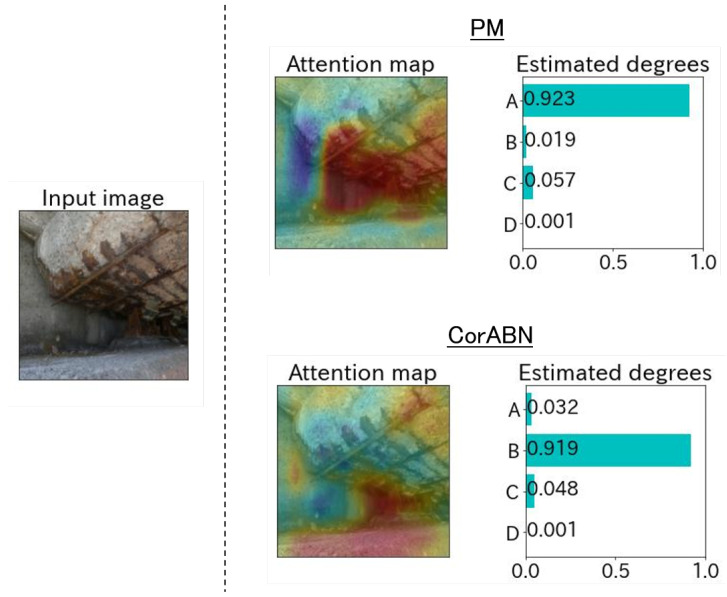
Estimated example at the degradation degree A for the distress type of rebar corrosion.

**Figure 6:**
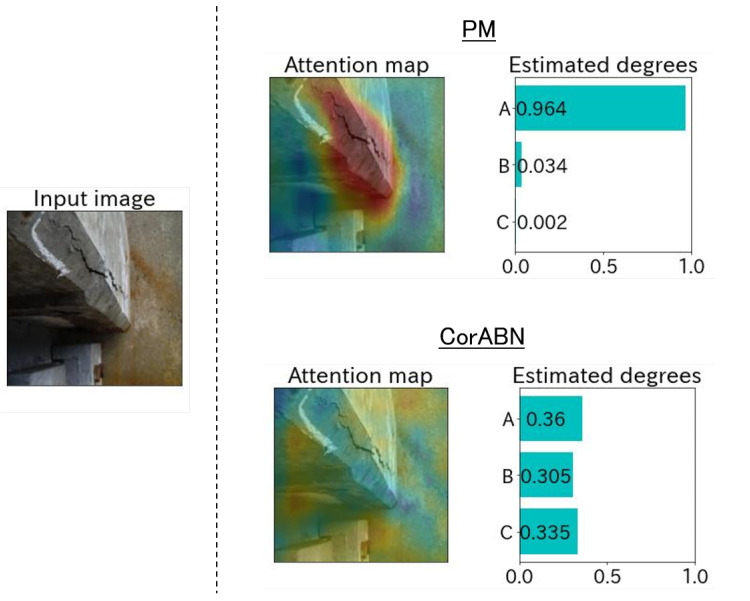
Estimated example at the degradation degree A for the distress type of concrete scaling.

**Figure 7:**
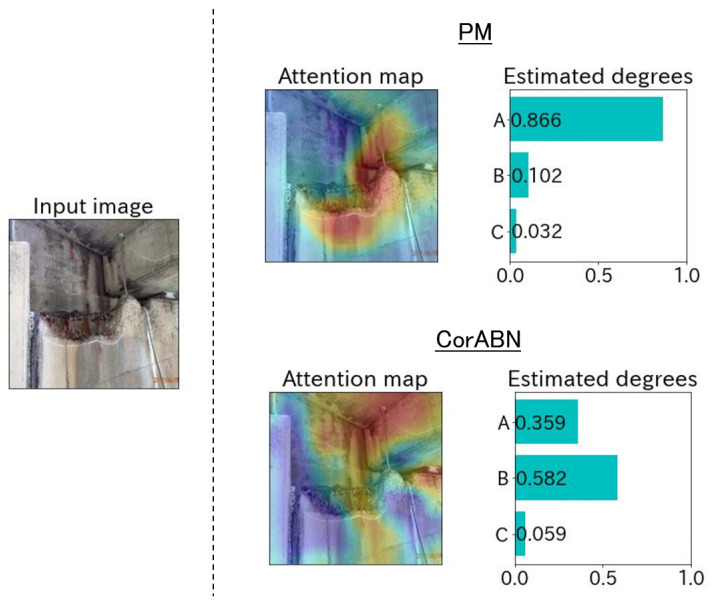
Estimated example at the degradation degree A for the distress type of concrete spalling.

**Figure 8:**
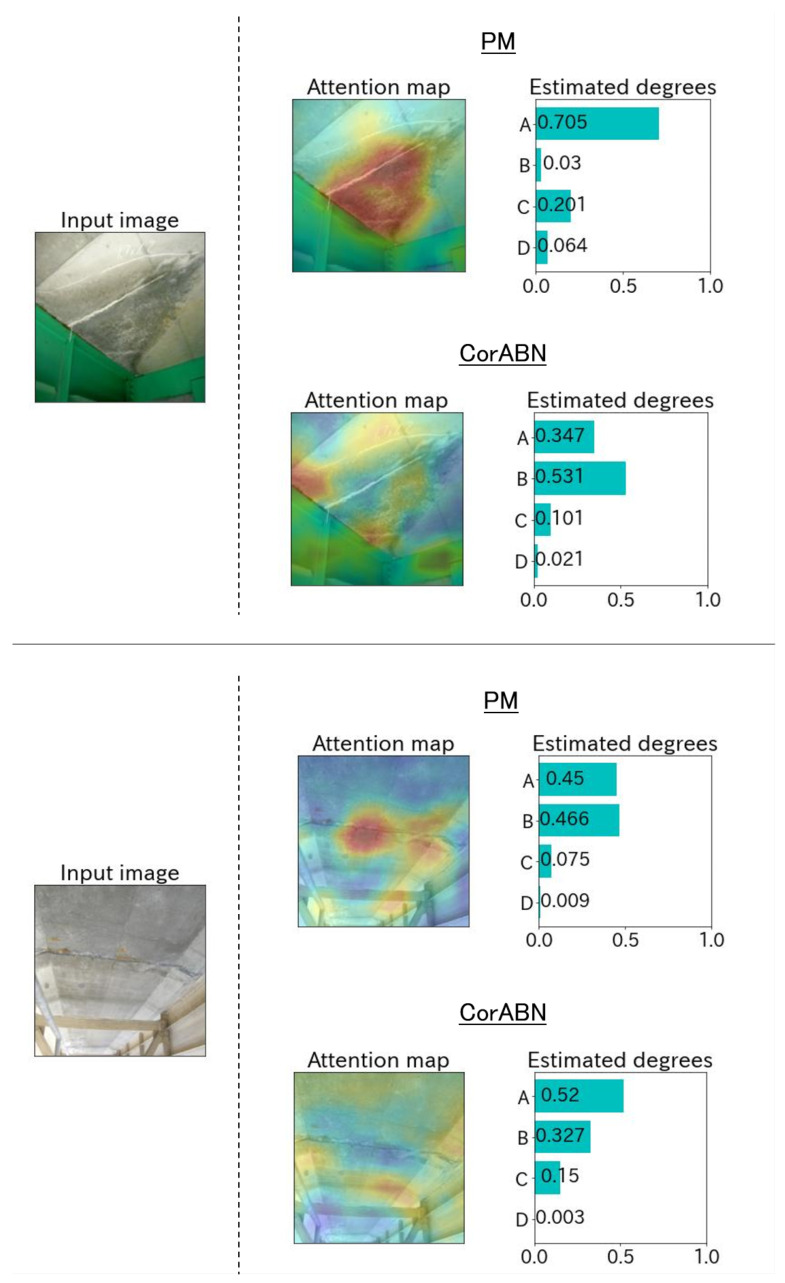
Estimated examples at the degradation degree A for the distress type of efflorescence.

Figure 4, Figure 5, Figure 6, Figure 7 and Figure 8 show the qualitative evaluation results of each distress in PM and CorABN [8]. The difference between PM and CorABN is the presence or absence of using the representation obtained through contrastive learning. In Figure 4, Figure 5, Figure 6, Figure 7 and Figure 8, the results are shown at the most urgent degradation degree A for each distress type. The attention maps of PM focus on the distress regions more appropriately compared to CorABN. In particular, the attention maps of PM appropriately display the regions of distress in red, while those of CorABN highlight the regions that are either too large, insufficient, or irrelevant to the distress. Figure 4, Figure 5, Figure 6 and Figure 7 illustrate the class probabilities of PM that exceeds those of CMs in the degradation degree estimation. Figure 8 presents the two qualitative results of efflorescence. In the upper part of the two results, PM appropriately generates the attention map and classifies the degradation degree. In the lower part, PM calculates the asymptotic probabilities for degradation degrees A and B, while CorABN achieves a slightly higher performance. However, a significant difference is found between PM and CorABN for the generated attention maps. PM highlights the distress regions more appropriately, while CorABN does not highlight the region. The results suggest that PM may perform the degradation degree estimation based on a better understanding of the location of the distress regions in the images. In terms of supporting engineers’ determination of the degradation degrees, these results revealed the utility of PM that can appropriately show the reasons for the estimated degradation degree. The qualitative evaluations for the various distresses suggest that PM can effectively classify the distress images for urgent repair.

### 4.3. Discussions

We provide discussions of the relationship between the estimation performance and the equilibrium of samples per degradation degree in training data used for supervised learning. First, we compare the performance of PM with that of CorABN. The ranges of improvement in the three types of distresses classified by the four-stage degradation degrees (i.e., crack, efflorescence, and rebar corrosion) are significantly different. Between PM and CorABN, the improvements of the averages of the four degrees for the rebar corrosion are greater than those for crack and efflorescence as shown in Table 6, Table 7 and Table 8. In particular, degree A of rebar corrosion archives clear improvement as shown in Table 8. Rebar corrosion was composed of an unbalanced dataset with a small number of samples in degree A as shown in Table 1, Table 2 and Table 3. Therefore, the prior representation of PM is confirmed as useful for supporting the estimation of the degradation degree in the case of a small dataset. In Table 8, the other degrees B, C, and D are largely improved compared to crack and efflorescence. Next, we compare the performances of PM and CorABN with those of the other CMs. As regards rebar corrosion, for the degradation degree A, ABN and most CNNs can hardly classify it, while PM and CorABN improve the performances in Table 8. Using the text data is effective in estimating the degradation degree with training data of small sample sizes. The results suggest the suitability of using CorABN as the classification model in PM. PM validity was confirmed for an unbalanced dataset.

A comparison of all distress types shows that the highest estimation performance is crack. The type of crack in Table 1 was composed of the largest amount of data for supervised learning in the experiment. The performances may be improved by increasing the amount of training data in supervised learning for other distresses. However, this approach would increase the burden on engineers in terms of annotation. PM can effectively improve the performance by using unlabeled data without increasing the amount of data for supervised learning, in which it is possible to utilize the contrastive learning representation for the classification model of distress images.

Our study shows the findings that unlabeled distress images in road infrastructures can be used in degradation degree estimation by constructing a CNN-based model through contrastive learning. It has been reported that the introduction of convolution layers is effective in extracting features from distress images of infrastructures [5,6,7,24,25,26,27,28,29,30], not only in degradation degree estimation. The PM’s approach based on contrastive learning can be applied to other tasks in civil engineering. Our findings are highly versatile for proposing new deep learning methods for infrastructures and can contribute to accelerating progress in this field.

The studies in civil engineering [5,6,7,8,9,10,24,25,26,27,28,29,30] are motivated by real-world applications, and the position of our paper is the same. With the introduction of contrastive learning, it is possible to estimate the degradation degree by using distress images simply taken from the real world without annotation. In the future, it is expected that advanced techniques, such as drones, will make it easy to take distress images on road infrastructure and a large number of distress images will become available. Additionally, with the background that degradation in road infrastructures is a serious problem worldwide, our study contributes to proposing a framework transcending national governments and management organizations. The assigned label information including degradation degrees is different for each management organization of road infrastructures. Therefore, we believe that our method can contribute to solving the global issue in the situation of not assigning uniform labels.

In this paper, we especially aim to assist engineers in the judgment of the degradation degrees to distress images. In most cases, the final judgment of the degradation degree of one distress image requires decisions by multiple specialized experts. An automatic degree estimation technique enables engineers to make the final decision based on the results of the technique. In particular, our ABN-based method is used for the degradation degree estimation by calculating an attention map. It is possible to show the reason for the classification by highlighting the important regions in the distress image and assist the engineers by referring to the attention map when they make the final judgment.

### 4.4. Limitation and Future Work

This section provides a limitation and some future works. We use some small datasets consisting of pairs of distress images and labels for the supervised learning in the classification model of PM. However, this label information is not fully utilized in the contrastive learning of PM. For example, if there are two distress images assigned with the same distress type and degradation degree, a total of four views can be generated from the targets by image transformations. In contrastive learning, the latent space is constructed so that views from the same target are embedded to be close to each other. On the other hand, views with different original targets are learned to be far away from each other, even though both targets are assigned with the same degradation degree. Since the final task is the estimation of degradation degree, it is desirable for views from targets with the same degree to be embedded close to each other when constructing the latent space. We believe that a clue for solving this problem is the supervised contrastive learning approach which has been proposed in the study [46].

In this study, PM introduces unsupervised contrastive learning regardless of labels, and it can construct the model across distress images of multiple datasets. However, the crossing approach conflicts with the supervised contrastive learning [46] due to the necessity of uniform labels. It is difficult to assume a situation that distress images of each dataset are assigned labels based on the same criteria. Therefore, we will solve this problem by assigning pseudo labels. This approach enables degradation degree estimation of distress images by introducing supervised contrastive learning, even across multiple datasets.

Finally, we provide a consideration of the improvement of the model itself. Vision Transformer has been attracting attention in the computer vision field. Vision Transformer-based methods [47,48] can reportedly achieve a higher performance than CNN-based methods for image classification in general object recognition. Thus, we are considering changes to the common backbone of contrastive learning and image classification models to a Transformer-based one.

## 5. Conclusions

In this paper, we proposed a method for distress image classification in road infrastructures using the prior representation obtained through contrastive learning, which can use unlabeled images. The proposed method obtained the parameters of a deep learning model obtained from distress images by contrastive learning as the prior representation of road infrastructures. These parameters were introduced to our classification model to improve the performance of the degradation degree estimation. We achieved the use of unlabeled images for estimating degradation degrees of distress images. We also confirmed the effectiveness of the proposed method for multiple types of distresses observed from real-world road infrastructures. The experimental findings can provide a labor-saving clue for inspection work on aging road infrastructures.

## Figures and Tables

**Table 1:** Number of images in the distress type of crack.

Degradation Degree	Training	Validation	Test	Total
A	1915	228	227	2370
B	2085	237	265	2587
C	1897	233	251	2381
D	2102	265	277	2644

**Table 2:** Number of images in the distress type of efflorescence.

Degradation Degree	Training	Validation	Test	Total
A	1039	143	141	1323
B	1125	150	152	1427
C	1096	143	129	1368
D	842	105	119	1066

**Table 3:** Number of images in the distress type of rebar corrosion.

Degradation Degree	Training	Validation	Test	Total
A	210	19	35	264
B	1639	234	185	2058
C	1236	164	146	1546
D	1002	115	133	1250

**Table 4:** Number of images in the distress type of concrete scaling.

Degradation Degree	Training	Validation	Test	Total
A	1503	190	203	1896
B	1333	171	165	1669
C	1086	132	152	1370

**Table 5:** Number of images in the distress type of concrete spalling.

Degradation Degree	Training	Validation	Test	Total
A	1326	168	179	1673
B	1307	163	166	1636
C	1056	127	133	1316

**Table 6:** F1-scores of PM and CMs in the distress type of crack. The best scores for each degree and the average are presented in bold.

	Degree: A	Degree: B	Degree: C	Degree: D	Average
**PM**	**0.781**	**0.634**	**0.617**	**0.719**	**0.688**
CorABN (ImageNet) [8]	0.776	0.627	0.599	0.704	0.676
ABN [12]	0.741	0.561	0.511	0.661	0.619
ResNet50 [38]	0.746	0.556	0.487	0.651	0.610
SeNet154 [41]	0.730	0.572	0.527	0.650	0.620
DenseNet121 [43]	0.695	0.490	0.436	0.608	0.557
InceptionV4 [42]	0.733	0.527	0.452	0.633	0.586
EfficientNetB5 [44]	0.712	0.552	0.500	0.653	0.604

**Table 7:** F1-scores of PM and CMs in the distress type of efflorescence. The best scores for each degree and the average are depicted in bold.

	Degree: A	Degree: B	Degree: C	Degree: D	Average
**PM**	0.699	**0.532**	0.473	**0.715**	**0.605**
CorABN (ImageNet) [8]	**0.707**	0.516	**0.497**	0.684	0.601
ABN [12]	0.602	0.480	0.327	0.592	0.500
ResNet50 [38]	0.586	0.445	0.293	0.576	0.475
SeNet154 [41]	0.610	0.489	0.404	0.607	0.528
DenseNet121 [43]	0.413	0.381	0.284	0.494	0.393
InceptionV4 [42]	0.517	0.403	0.306	0.544	0.443
EfficientNetB5 [44]	0.605	0.489	0.367	0.618	0.520

**Table 8:** F1-scores of PM and CMs in the distress type of rebar corrosion. The best scores for each degree and the average are presented in bold.

	Degree: A	Degree: B	Degree: C	Degree: D	Average
**PM**	**0.449**	**0.677**	**0.468**	**0.579**	**0.543**
CorABN (ImageNet) [8]	0.311	0.647	0.449	0.543	0.487
ABN [12]	0.133	0.600	0.436	0.438	0.402
ResNet50 [38]	0.020	0.611	0.456	0.437	0.381
SeNet154 [41]	0.169	0.615	0.467	0.475	0.431
DenseNet121 [43]	0.000	0.592	0.336	0.287	0.304
InceptionV4 [42]	0.000	0.581	0.294	0.199	0.269
EfficientNetB5 [44]	0.268	0.606	0.439	0.491	0.451

**Table 9:** F1-scores of PM and CMs in the distress type of concrete scaling. The best scores for each degree and the average are depicted in bold.

	Degree: A	Degree: B	Degree: C	Average
**PM**	**0.632**	**0.399**	**0.492**	**0.507**
CorABN (ImageNet) [8]	0.630	0.370	0.402	0.467
ABN [12]	0.573	0.338	0.180	0.364
ResNet50 [38]	0.558	0.359	0.173	0.364
SeNet154 [41]	0.599	0.329	0.392	0.440
DenseNet121 [43]	0.542	0.261	0.023	0.275
InceptionV4 [42]	0.553	0.374	0.082	0.337
EfficientNetB5 [44]	0.574	0.340	0.425	0.446

**Table 10:** F1-scores of PM and CMs in the distress type of concrete spalling. The best scores for each degree and the average are presented in bold.

	Degree: A	Degree: B	Degree: C	Average
**PM**	**0.644**	**0.491**	**0.556**	**0.563**
CorABN (ImageNet) [8]	0.616	0.461	0.510	0.529
ABN [12]	0.567	0.370	0.389	0.442
ResNet50 [38]	0.559	0.358	0.340	0.419
SeNet154 [41]	0.608	0.434	0.416	0.486
DenseNet121 [43]	0.503	0.377	0.271	0.383
InceptionV4 [42]	0.554	0.342	0.414	0.437
EfficientNetB5 [44]	0.568	0.427	0.372	0.456

**Table 11:** F1-scores of eight methods for rebar corrosion. A total of seven PMs is composed of the six cases of introducing the parameters of contrastive learning to some modules in CorABN and the case of all modules. Each method’s name contains the module that introduced the parameters of contrastive learning. FME, AMG, and CF correspond to the feature map extractor, attention map generator, and classifier, respectively. In addition, we used CorABN using ImageNet parameters following the study [8]. The best scores for each degree and the average are presented in bold.

	Degree: A	Degree: B	Degree: C	Degree: D	Average
PM (all module)	**0.449**	**0.677**	0.468	**0.579**	**0.543**
PM (FME)	0.377	0.666	**0.472**	0.557	0.518
PM (AMG)	0.265	0.661	0.434	0.547	0.477
PM (CF)	0.267	0.655	0.457	0.541	0.480
PM (FME + AMG)	0.396	0.676	0.466	0.571	0.527
PM (AMG + CF)	0.294	0.656	0.440	0.545	0.484
PM (FME + CF)	0.409	0.674	0.459	0.565	0.527
CorABN (ImageNet) [8]	0.311	0.647	0.449	0.543	0.487

**Table 12:** F1-scores of PM using SimCLR and BYOL parameters and CorABN using ImageNet parameters. The best scores for each degree and the average are presented in bold.

	Degree: A	Degree: B	Degree: C	Degree: D	Average
Crack					
PM (SimCLR)	0.781	0.634	**0.617**	**0.719**	**0.688**
PM (BYOL)	**0.782**	**0.642**	0.603	0.704	0.683
CorABN (ImageNet) [8]	0.776	0.627	0.599	0.704	0.676
Efflorescence					
PM (SimCLR)	0.699	0.532	0.473	**0.715**	0.605
PM (BYOL)	**0.714**	**0.543**	**0.504**	0.713	**0.618**
CorABN (ImageNet) [8]	0.707	0.516	0.497	0.684	0.601
Rebar corrosion					
PM (SimCLR)	**0.449**	**0.677**	0.468	**0.579**	**0.543**
PM (BYOL)	0.324	0.663	**0.473**	0.554	0.504
CorABN (ImageNet) [8]	0.311	0.647	0.449	0.543	0.487
Concrete scaling					
PM (SimCLR)	**0.632**	0.399	**0.492**	–	**0.507**
PM (BYOL)	0.618	**0.428**	0.336	–	0.461
CorABN (ImageNet) [8]	0.630	0.370	0.402	–	0.467
Concrete spalling					
PM (SimCLR)	**0.644**	**0.491**	**0.556**	–	**0.563**
PM (BYOL)	0.619	0.484	0.515	–	0.539
CorABN (ImageNet) [8]	0.616	0.461	0.510	–	0.529

**Table 13:** F1-scores of PM using SimCLR parameters corresponding to multiple dimensions of the feature representation. The best scores for each degree and the average are presented in bold.

	Degree: A	Degree: B	Degree: C	Degree: D	Average
Crack					
PM ($d_{r}=64$)	**0.781**	0.634	**0.617**	0.719	0.688
PM ($d_{r}=128$)	**0.781**	**0.644**	0.614	**0.721**	**0.690**
PM ($d_{r}=256$)	0.779	0.637	0.609	0.713	0.684
Efflorescence					
PM ($d_{r}=64$)	0.699	0.532	0.473	**0.715**	**0.605**
PM ($d_{r}=128$)	0.703	**0.535**	0.469	0.683	0.597
PM ($d_{r}=256$)	**0.709**	0.531	**0.487**	0.685	0.603
Rebar corrosion					
PM ($d_{r}=64$)	0.449	0.677	0.468	0.579	0.543
PM ($d_{r}=128$)	0.473	**0.686**	0.465	0.566	0.548
PM ($d_{r}=256$)	**0.515**	0.682	**0.498**	**0.586**	**0.570**
Concrete scaling					
PM ($d_{r}=64$)	0.632	0.399	0.492	–	0.507
PM ($d_{r}=128$)	0.642	**0.418**	0.486	–	**0.515**
PM ($d_{r}=256$)	**0.650**	0.389	**0.505**	–	0.514
Concrete spalling					
PM ($d_{r}=64$)	**0.644**	0.491	0.556	–	0.563
PM ($d_{r}=128$)	0.619	0.495	0.551	–	0.555
PM ($d_{r}=256$)	0.643	**0.515**	**0.557**	–	**0.571**

## Data Availability

Experimental data cannot be disclosed.
